# Identification of Essential Proteins Based on Ranking Edge-Weights in Protein-Protein Interaction Networks

**DOI:** 10.1371/journal.pone.0108716

**Published:** 2014-09-30

**Authors:** Yan Wang, Huiyan Sun, Wei Du, Enrico Blanzieri, Gabriella Viero, Ying Xu, Yanchun Liang

**Affiliations:** 1 Key Laboratory of Symbolic Computation and Knowledge Engineering of Ministry of Education, College of Computer Science and Technology, Jilin University, Changchun, China; 2 Department of Information and Communication Technology, University of Trento, Povo, Italy; 3 Institute of Biophysics, National Research Council, University of Trento, Povo, Italy; 4 Computational Systems Biology Lab, Department of Biochemistry and Molecular Biology and Institute of Bioinformatics, University of Georgia, Athens, GA, United States of America; London, United Kingdom

## Abstract

Essential proteins are those that are indispensable to cellular survival and development. Existing methods for essential protein identification generally rely on knock-out experiments and/or the relative density of their interactions (edges) with other proteins in a Protein-Protein Interaction (PPI) network. Here, we present a computational method, called EW, to first rank protein-protein interactions in terms of their *Edge Weights*, and then identify sub-PPI-networks consisting of only the highly-ranked edges and predict their proteins as essential proteins. We have applied this method to publicly-available PPI data on *Saccharomyces cerevisiae* (*Yeast*) and *Escherichia coli* (*E. coli*) for essential protein identification, and demonstrated that EW achieves better performance than the state-of-the-art methods in terms of the precision-recall and Jackknife measures. The highly-ranked protein-protein interactions by our prediction tend to be biologically significant in both the *Yeast* and *E. coli* PPI networks. Further analyses on systematically perturbed *Yeast* and *E. coli* PPI networks through randomly deleting edges demonstrate that the proposed method is robust and the top-ranked edges tend to be more associated with known essential proteins than the lowly-ranked edges.

## Introduction

Essential proteins are indispensable for the survival of an organism under certain conditions [1]. Reliable identification of essential proteins can contribute to the understanding of the key biological processes of an organism at a systems level, with significant implications to drug design, disease diagnosis and medical treatments. Experimentally, identifications of essential proteins are typically performed through gene knock-outs [1], [2] or RNA interference [3], coupled with examination of the viability of the affected organisms. Over the years, numerous proteins have been identified to be essential in a variety of organisms. Such data become particularly useful when used in conjunction with large-scale protein-protein interaction (PPI) data collected using high-throughput techniques such as the yeast-hybrid technique [4]. As of now, a number of PPI networks have been constructed for *Saccharomyces cerevisiae* (*Yeast*) [4], *Escherichia coli* (*E. coli*) and *Caenorhabditis elegans* (*C. elegans*) [5], which have been organized into several PPI databases in the public domain, such as DIP [6], BioGRID [7], STRING [8] and MIPS [9].

A few studies have been published since 2000, aiming to establish relationships between experimentally-identified essential proteins and PPI networks. For example, Jeong *et al.* noted that the centrality of a protein in a PPI network, a property based on the network topology, is strongly related to the essentiality of the protein [10]. Similarly it has been demonstrated that proteins, that are interaction hubs in a PPI network tend to be essential as studies have shown that the deletion of a hub protein tends to be more lethal than deleting a non-hub protein in *Yeast*, *E. coli* and *C. elegans* [11]–[13]. Based on this observation, known as *centrality-lethality* rule [10], [14], numerous centrality-based measures for essential protein detection have been developed, such as the degree centrality [10], betweenness centrality [15], closeness centrality [16], subgraph centrality [17], eigenvector centrality [18], information centrality [19], network bottleneck [20], [21], and density of maximum neighbourhood component [22]. Basically these methods identify essential proteins by ranking them in terms of their centrality measures in a PPI network. In addition, a few edge-aided methods for analysing PPI networks PPI network have also been developed. For example, Radicchi *et al.* proposed the edge-clustering coefficient [23] for identifying essential proteins, considering both edge and node information. The *edge clustering coefficient centrality* (NC) is another edge-aided method [24], which employs the edge clustering coefficient concept to identify essential proteins in a PPI network. More recently, a number of studies have been published, which combine PPI networks with other biological information to further improve the prediction performance, mainly to overcome issues associated with both missing and false interactions in the existing PPI data. The group that developed the NC method recently proposed a strategy for constructing a weighted PPI network by considering gene-annotation information, which has enhanced the performance by edge-aided methods [25]. Further improvements were achieved through integration of gene-expression data (PeC) [26] and phylogenetic profile information (ION) [27] by the same group. Although the edge information plays an important role in the identification process, the above-mentioned methods fundamentally rank proteins according to the centrality measure in a PPI network.

In a different perspective, interactions among essential proteins have been taken into consideration and some researchers began to critically consider the traditional explanation of the observed centrality-lethality relationship to propose different points of view. In 2005, Pereira-Leal *et al*. [28] pointed out that essential proteins tend to be more frequently connected with other essential proteins rather than to non-essential proteins in *Yeast* PPI networks. After removing all the non-essential proteins from a PPI network, they observed that approximately 97% of the essential proteins are still connected to each other, suggesting a tight relationship among essential proteins. In 2006, He *et al*. [14] reconsidered the reason why highly connected nodes tend to be essential, and proposed the concept of essential protein-protein interactions. They argued that the essentiality of proteins derives from the essentiality of protein-protein interactions. This new viewpoint about essentiality raises an issue about how to verify the essentiality of interactions. Some edge-aided methods that combine PPI networks with other biological information suggested that edges between two proteins are related to the essentiality of proteins [24]–[27]. A number of computational approaches have been developed to score the relatedness of proteins connected by edges in a PPI network. Some of these measures are based on associations between two proteins obtained by Gene Ontology [25], gene co-expression [26], number of triangles an edge belongs (NTE) [24] and pairwise sequence similarities [28], [29].

In this paper, we present a novel strategy for essential protein identification based on *edge weights* (EW) for ranking protein-protein interactions within a PPI network. EW scores the importance of an edge in the network by combining several widely used PPI topological information and biological measures. Then it ranks the edges according to their weights and predicts essential proteins based on identification of sub-networks consisting of only highly ranked edges. Our application of EW on *Yeast* and *E. coli* PPI data for essential protein prediction demonstrated that it achieves better performance than the state-of-the-art methods. Our predicted essential protein-protein interactions tend to be more biologically significant in both the *Yeast* and *E. coli* PPI networks. Its performance on systematically perturbed *Yeast* and *E. coli* PPI networks through randomly deleting edges demonstrates that the proposed method is robust and the top-ranked edges tend to be more associated with known essential proteins than low ranked edges.

## Material and Methods

### Data Source

#### 1. Protein-Protein Interaction Network and Essential Proteins List

PPI data of *Yeast* were downloaded from the DIP [6] database (release of Oct. 18^th^, 2012). The dataset consists of 22,061 distinct interactions among 4,979 proteins. The list of essential genes of *Yeast* is collected from the OGEE [30] database, which groups all genes into three categories: essential, non-essential and conditional when the essentiality status of the gene varies in different environments. In our analysis, we consider conditional genes as essential because interaction-based methods identify essential genes in different special conditions represented by PPIs and find out also conditionally essential genes. Overall, the *Yeast* network consists of 1,209 essential proteins, 3,322 nonessential ones, and 448 unknown proteins that are in DIP but not in OGEE.

Similarly, we downloaded PPI data of *E. coli* from DIP [6] and the essential genes from OGEE [30]. Over 1,000 protein-protein interactions are either single-pair interactions or part of small and unconnected networks with fewer than five nodes. We removed them from our analyses. At the end, the cleaned-up *E. coli* PPI network consists of 2,528 proteins and 11,496 interactions. Out of these proteins, 444 are essential, 1,403 are nonessential, and 671 unknown ones.

#### 2. Gene Expression Data

Gene-expression dataset GSE3431 of *Yeast* [31] was downloaded from GEO [32], which was collected during three successive metabolic cycles, with 12 time points in each cycle, ∼25 minutes apart. The dataset contains 36 samples with 6,777 genes of which 4,858 are involved in the aforementioned *Yeast* PPI network.

*E. coli* gene expression data GSE6425 [33] was also downloaded from GEO, which has expression data of two *E. coli* strains, MG1655 and UTI89, harvested at multiple time points during aerobic or anaerobic growth in Luria-Bertani medium. We used the MG1655 data which contains 22 samples with 4,345 genes.

The detailed information of these data is given in File S1 through File S4.

### Ranking Edges of PPI Networks to get Essential Protein List

**Figure 1:**
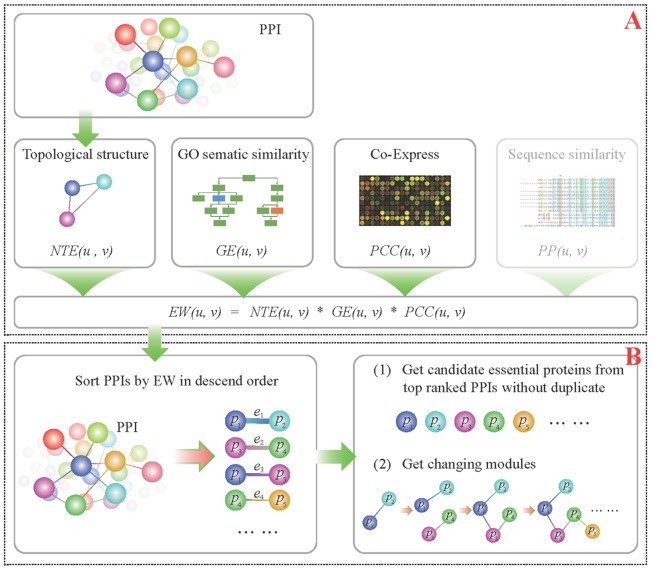
The whole workflow of EW method. (**A**) Edge Weight Computation; (**B**) Essential Protein Identification based on Edge Weights.

The whole workflow of EW method is easy to implement as shown in Figure1. We firstly calculate the weights of each edge by multiple several measures of protein pairs, and then sorted the weights to get the essential protein candidates list.

#### 1. Edge Weight Computation

We use the following four commonly used measures for evaluating the relationship between two proteins, whose nodes are connected by an edge in a PPI network: GO functional similarity (GE) [34], co-expression levels among genes (PCC) [26], the number of times that a PPI pair involved in PPI triangles (NTE) [24], and the protein-protein sequence similarity measured using the Jukes-Cantor likelihood (PP) [35] (see File S7). The edge weight 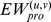 between proteins *u* and *v* is computed as
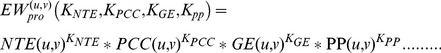
(1)where 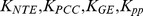 are scaling parameters set to 0 or 1, representing whether the corresponding measure is used in the 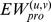 calculation or not. Here we simply use multiplication process to combine these measures, because the value ranges of these measures are different, even the same measures between different species. In fact, the addition or normalization processes may influence the final edge order, and therefore the final list of essential proteins. On the other hand, the multiplication process may keep the original feature values and is more generalization across different species. Furthermore, in the pre-test, we have compared the read out of addition and multiplication, finding that the latter is the easiest and best approach to get good performance.

In a preliminary analysis on *Yeast* for the effectiveness of the parameters of formula (1) (Figure S1 and S2), PP had virtually no effect on the accuracy identification of essential proteins, and was therefore removed from our consideration. This consideration gives rise to the following edge weight 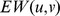 revised formula (2) (see Figure 1A).
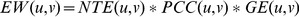
(2)


#### 2. Essential Protein List based on Edge Weights

After calculating each edge weight in a PPI network, we sort the edges in descending order of their weights (see Figure 1B). Let 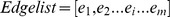 be the sorted list, where 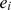 is the *i-*th edge in the list with 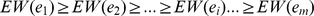. For each edge, we create a list containing two proteins connected by the edge. And we generate a list of ranked proteins 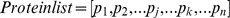, in such a way that protein 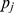 precedes protein 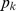 in the list if and only if 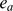 is the first edge where 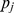 appears and 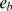 is the first edge where 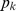 appears and 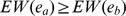. By choosing a different *k* value, we can get the top *k* essential protein candidates. For example in Figure 1B, *Edgelist  = [e1,e2,e3,e4…]* was obtained after sorting their edge weights in descending order. We extract edges one by one from the *Edgelist* according to the order, and in each extract process, we put the two nodes belongs to the edge into candidate proteins set sequentially if they don't appear in this set before. *e1* was the first chosen edge, and then its two nodes *p1* and *p2* was set into candidate proteins set. When *e3* was chosen, we found *p1* and *p3* had appeared in candidate protein set, so in this extraction process, there was not new protein comer. In a similar way, we added all the edges' nodes into protein set without duplicate.

### Performance Evaluation

It has been established that NC, PeC and ION methods perform better than the previously published centrality-based measures [24], [26], [27]. Therefore, in our performance assessment we compare EW against these methods. To evaluate the overall performance, we use the precision-recall, and Jackknife curves as presented in [36], which measure the number of true positives among the top ranked list. In addition, we assesse EW's performance on perturbed PPI networks to assess the robustness of each method, and analysed pathway enrichment by DAVID to examine the biological functions of the obtained protein modules [37].

#### 1. Precision-Recall Curve

A Precision–Recall (PR) curve is obtained by plotting: 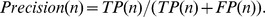


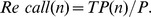
where *TP(n)* is the number of true positives among the top *n* ranked proteins, and *FP(n)* is the number of non-essential proteins incorrectly predicted as essential among the top *n* ranked proteins, and *P* is the total number of essential proteins under consideration.

#### 2. Jackknife Curve

We use the Jackknife curve [36] to assess the generality of our trained predictor. A Jackknife curve represents the number of samples that are correctly predicted among a top ranked prediction list, denoted as 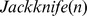 for the number of true positives among the top n predictions. In a 2D representation, the x-axis denotes the number of proteins sorted in a descending order while the y-axis represents the number of essential proteins correctly predicted among the top n predictions, with n being a number along the x-axis. When doing performance comparison, the EW's Jackknife curve plots the number of essential proteins, namely *TP(n)* (y-axis) against the length of the lists, namely n (x-axis).

#### 3. Pathway Enrichment Analysis

We have carried out a pathway enrichment analysis among the top ranked predictions, using DAVID along with statistical significant p-values calculated using a modified Fisher's exact test [37], [38]. To correct the enrichment p-values and to control the family-wide false discovery rate (FDR), a *Benjamini Hochberg* (BH) testing correction is used by DAVID.

#### 4. Robustness Test

To assess the robustness of the EW method, we have perturbed the original PPI data by deleting X edges, for X = 100, 500 and 1,000, from the top, the bottom and randomly in the ranked EW edge list, respectively. We then applied EW, PeC and NC on these perturbed networks and observed the changes in identification read out.

## Results

### EW Performance on *Yeast* and *E. coli*

#### 1. Comparison on *Yeast* PPI data

**Figure 2:**
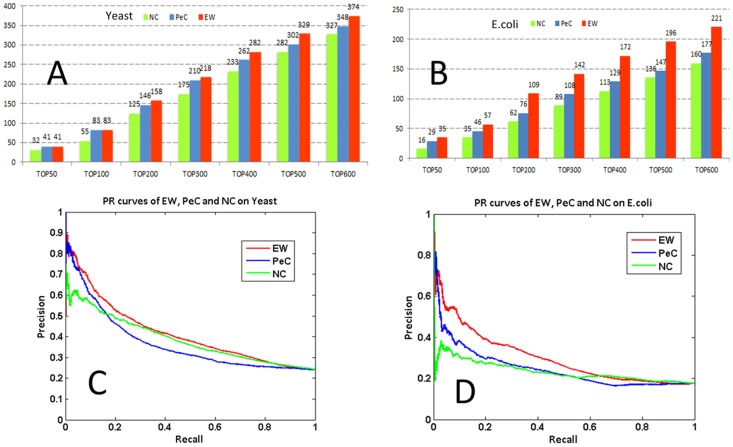
EW performance compare with NC and PeC methods. (**A**)(**B**): Comparison among the numbers of essential proteins identified by EW, NC and PeC on *Yeast* and *E.coli*, respectively, when selecting 50, 100, 200, 300, 400, 500 and 600 top ranked proteins; (**C**)(**D**): PR curves of EW, NC and PeC on *Yeast* and *E.coli*.

We compared EW with NC and PeC in terms of number of essential proteins among their top-ranked proteins (Figure 2A), and found that EW performs substantially better than the other two programs on the *Yeast* dataset described in the Methods section. Specifically, among the top 100, 200 and 600 ranked predictions, EW correctly predicts 83, 158 and 374 essential proteins and in comparison, NC correctly predicts 55, 125 and 327 proteins, and PeC correctly predicts 83, 146 and 348 verified essential proteins, respectively. For this prediction, we used the default parameters of each programs.

We also compared the three programs measured using the precision-recall and Jackknife curves, and found that EW consistently outperforms PeC and NC, as shown in Figure 2C and Figure S3A.

Some proteins are predicted to be essential by the DIP database but are unknown proteins in the OGEE database. We noted that at least two of such unknown proteins are among our top predictions, SMX2 and TRA1. SMX2 ranks as the top 186^th^ prediction by EW, and was reported as an essential protein by Guri *et al*. [1]. TRA1 is ranked number 300 among EW's prediction, and was found to be essential by Saleh *et al*. [39].

#### 2. Comparison on *E. coli* PPI data

EW shows substantially better performance than NC and PeC on the *E. coli* PPI data (see Methods section). Among the top 100, 200, 400 and 600 predictions, EW correctly predicts 57, 109, 172 and 221 verified essential proteins. In comparison, NC correctly predicts 35, 62, 113 and 160, and PeC correctly predicts 46, 76, 129 and 177. The detailed data for this comparison is given in Figure 2B. Similar results are observed in terms of precision-recall and Jackknife curve as illustrated in Figure 2D and Figure S3B.

Similar to what observed in *Yeast*, we found a number of *E. coli* proteins not included in OGEE, which were reported to be essential proteins in the literature and are predicted by our program. For example, FUSA, which is ranked as the 3^rd^ protein by EW, was reported to be an essential protein according to the DEG database (DEG10040515) and in Baba, *et al*. [40].

Finally, we compared the predictions of EW against ION. The NC's performance curves on the tested dataset are almost the same of those shown in Figure 2C, 2D and in the ION paper [27]. Though the shape of the PR curve is slightly better than NC on *Yeast*, ION's performance has low precision at low recall values on the *E. coli* dataset, while different from the performance on *Yeast*. In comparison, EW and PeC display high precisions at low recall values, similarly to the performance on *Yeast* shown in Figure 2C, and EW has even better performance than PeC. The high-level of performance on the two very different datasets indicates that EW is generally stable.

### Importance of Top Edges Found by EW and Its Robustness

**Figure 3:**
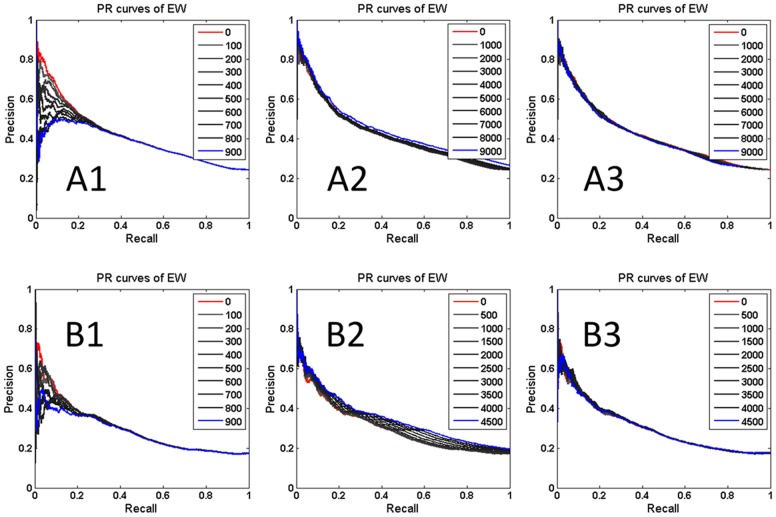
PR curves of EW methods on the perturbed *Yeast* and *E.coli* PPI networks for essential protein identification. The PPI networks are perturbed from the top, the bottom and randomly by deleting X edges in 10 steps in the ranked EW edge list. (**A1, B1**) From the top for X = 100 on *Yeast* and *E. coli*; (**A2, B2**) Form the bottom for X = 1000 and 500 on *Yeast* and *E. coli*, respectively; (**A3, B3**) For X = 1000 and 500 randomly on *Yeast* and *E. coli*, respectively.

In order to study the importance of the edges found with EW and the robustness of the method, we run EW on a series of perturbed PPIs as shown in Figure 3.

Perturbations on the *Yeast* PPI was obtained by deleting 100 edges in 10 steps from the top edges in the ranked edge list of EW (see Figure 3A1). We observed that the edge removal through each step substantially degrades the prediction results in all the methods. We further noted that deleting 5% from the top of all ranked edges, the essential-protein prediction can significantly change.

In comparison, when deleting edges from the PPI chosen the bottom of the ranked edge list, e.g. by deleting 1000 edges each step, each deletion step did not change our essential protein prediction. The same happened when deleting 10,000, almost half of the edges of the whole PPI network. These results indicate that the edge ranking list by EW indeed capture the key information associated with essential proteins.

Similar results were achieved on the *E. coli* PPI as shown in Figure 3B1–3B3.

When applying the above analyses to the PeC and NC methods, we found that the top proteins identified by EW are also more important than the bottom proteins of the ranked edge list, which may influence PeC and NC performance more (Figure S4 and S5).

### Case studies on selected clusters with high-ranking genes in PPI Networks

**Figure 4:**
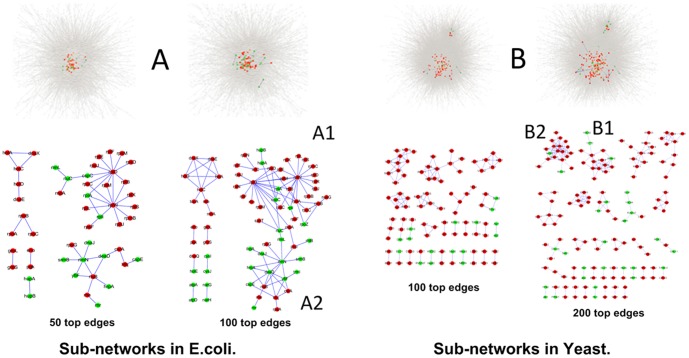
Sub-networks in (A) *E.coli* and (B) *Yeast* PPI network formed by top edges. The red nodes are essential proteins and the green ones are non-essential proteins.

It is difficult to give a fixed cut-off value for the final candidate list. The final number of top proteins selected for further analysis depends on related available information and on the overall capabilities that a lab can deploy for a particular research purpose. We examined the sub-networks consisting of only high-ranking edges in a PPI network, each of which is termed as a network *module*. We use Cytoscape (version 2.8.0) [41] to show the identified modules in Figure 4, and list of proteins of modules in Figure 4 are shown in File S6.

We carried out pathway enrichment analyses of each network module consisting of only the top 100 edges of the *E. coli* PPI network; and of only the top 200 edges of the *Yeast* PPI network which found by EW (File S5 for the top protein lists on *E. coli* and *Yeast*, respectively). We found that genes belonging to network modules with at least ten proteins are generally involved in the same pathways or protein complexes, such as the identified modules A1 and A2 of *E. coli* and module B1 of *Yeast* have high Benjamini scores for the pathway-enrichment at 1.9E–47, 4.4E–10 and 5.3E–21, respectively (Table 1).

**Table 1:** Modular examples pathway enrichment analysis by DAVID.

Organism	Modular example	GO term	Count (%)	p-value	Benjamini
*Yeast*	B1	Proteasome	13 (87%)	5.3E–21	5.3E–21
	B2	–	–	–	–
*E.coli*	A1	Ribosome	27 (50%)	1.5E–49	1.9E–47
	A2	RNA degradation	7 (13%)	4.5E–11	4.4E–10

#### 1. *E. coli* modules

**Figure 5:**
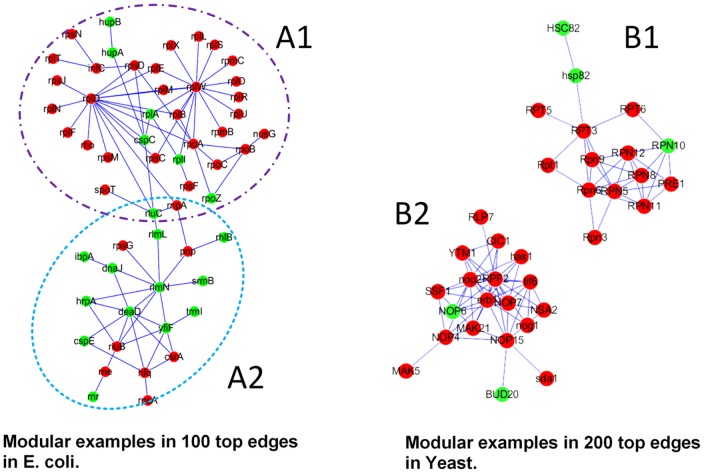
Modular examples in *Yeast* and *E.coli* PPI network by top edges. The red nodes are essential proteins and the green ones are non-essential proteins.

For the identified *E. coli* modules, module A1 consists of genes that encodes for ribosome related proteins and module A2 consists of proteins associated to ribosome biogenesis. All these proteins are related to post-transcriptional modification of RNA, in particular RNA methylation. Out of the 55 ribosomal genes in module A1, two major hubs appear connecting ribosomal proteins. The first hub is organized around RPL4 (rplD in Figure 5A1) which is a translational repressor and known to regulate the expression of the S10 operon by transcription attenuation[42]. The second hub is centered around RPL23 (rplW in Figure 5A1). As far as we know, this protein is not known to play specific roles in controlling the expression of other ribosomal proteins so it could be an interesting candidate for further investigation. Note that the transcripts of rpoS in A1 are targets of the cold shock protein C (CspC) which plays a role in the mRNA stability [43], and connects modules A1 and A2.

Module A2 consists mainly of non-essential proteins with a central hub in deadD. The protein has been demonstrated to restore the presence of both RPS1 and RPSS2 in ribosomes of the rpsB(ts) strain grown in non-permissive temperature, indicating its involvement in ribosome biogenesis [44]. Beside this protein, most of the interactions in Figure 5A2 involve enzymes relevant to RNA processing, which is increasingly recognized as potential sites of post-transcriptional regulation [45]–[48].

#### *2. Yeast* modules

Module B1(Figure 5B1) of *Yeast* consists of proteasome proteins and 13 out of 19 proteins of module B2(Figure 5B2) are involved in ribosome biogenesis [49]–[53].

Among the essential protein interactions in module B1 are those involving ATPases Rpt1-Rpt6 [54] and the non-ATPase proteasome subunits, Rpn1-3, Rpn5-13 and Rpn15 [55]. The central role of ribosome biogenesis and ribosome regulation found in *E. coli* is also found in the *Yeast data*. The B2 module not only represents the essential interactions involved in rRNA pre-processing but also covers connections with the cytoskeleton organization (such as actin depolarization, sda1 and microtubule association, YTM1), the translation initiation (tif6) and the proteolytic surveillance (CIC1). In addition, a number of nodes such as tif6 [56], NOP2P [57], NSA2 [58], and RPF2, which is a central node in module B2, are known to be essential players for processing of 27SB pre-rRNA.

## Conclusions

Unlike the centrality-based measures and edge-aided methods for essential protein identification, we proposed a method EW to rank protein-protein interactions in a PPI network through comparison of their edge weights, and identify essential proteins as connected sub-networks by top ranked edges. EW achieves better performance in terms of precision-recall and Jackknife measures than the state-of-the-art methods when applied to detection of essential proteins in both *Yeast* and *E. coli*. The analysis on perturbed PPI networks shows that our program also has higher prediction stability than the compared programs. We expect that the EW program will serve as a useful tool for identification of essential proteins in PPI networks of any organisms.

## Supporting Information

Figure S1
**PR curves of different combination of measures in EW on** ***Yeast*** **PPI networks.** 15 PR curves with different combination of 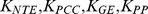 of 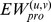 in formula (1) are illustrated. It can be seen that the results using the combination 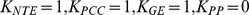, which is the top red line above all the others, has the best performance, which leads to 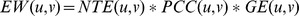 as the formula (2) for the EW method.(TIF)

Figure S2 **PR curves of different measures combination in EW without PP vs. with PP on Yeast PPI networks.** And PP vs. average performance by 10 times randomly sorted all edges on Yeast PPI networks. From the PR curves in (A)–(G), we can see that PP has virtually no effect on to the combination identification, except when combined with GE. In (H), the performance with PP alone is very similar to the PR curve performance of randomly sorted all edges (by 10 times average).
(TIFF)

Figure S3 **Jackknife curves of EW, NC and PeC on Yeast and E.coli PPI networks.** The yellow line whose slope is equal to the ratio between the total number of essential proteins and the total number of all the proteins is plotted as a baseline. It represents the expected performance of the probability for a random selection that how many essential proteins will randomly appear in a chosen protein list and it is used as a standard reference for comparison.
(TIF)

Figure S4 **PR curves of EW, PeC and NC methods on the perturbed** ***Yeast*** **PPI networks for essential proteins identification.** The *Yeast* PPI networks are perturbed from the top, the bottom and randomly by deleting X edges in 10 steps in the ranked EW edge lists. (A1, A4, A7) are the EW, PeC and NC performance of deleting edges from the top for X = 100; (A2, A5, A8) are the EW, PeC and NC performance of deleting edges from the bottom for X = 1000; (A3, A6, A9) are the EW, PeC and NC performance of deleting edges randomly for X = 1000.
(TIF)

Figure S5 **PR curves of EW, PeC and NC methods on the perturbed** ***E.coli*** **PPI networks for essential proteins identification.** The E. coli PPI networks are perturbed from the top, the bottom and randomly by deleting X edges in 10 steps in the ranked EW edge lists. (B1, B4, B7) are the EW, PeC and NC performance of deleting edges from the top for X = 100; (B2, B5, B8) are the EW, PeC and NC performance of deleting edges from the bottom for X = 500; (B3, B6, B9) are the EW, PeC and NC performance of deleting edges randomly for X = 500.
(TIF)

File S1 **Protein Information of Yeast, include protein uniprot ID and essential status.**
(XLS)

File S2 **Protein-Protein Interaction Information of Yeast, include protein pairs' uniprot IDs, GO semantics similarity, gene co-expression, number of triangles an edge belongs and pairwise sequence distance.**
(XLS)

File S3 **Protein Information of** ***E. coli*** **, include protein uniprot ID and essential status.**
(XLS)

File S4 **Protein-Protein Interaction Information of** ***E. coli*** **, include protein pairs' uniprot IDs, GO semantics similarity, gene co-expression and number of triangles an edge belongs.**
(XLS)

File S5 **Top protein list in Yeast (within top 200 edges) and** ***E. coli*** **(within top 100 edges).**
(XLS)

File S6 **Proteins list of modules A1, A2, B1 and B2 in** **Figure 4** **.**
(XLS)

File S7 **Measures in Edge Weight.**
(DOC)
