# Supplementary figures and images for: Identification of Essential Proteins Based on Ranking Edge-Weights in Protein-Protein Interaction Networks
Source: PLoS One. 2014 Sep 30;9(9):e108716. doi: 10.1371/journal.pone.0108716 (PMC4182551; doi:10.1371/journal.pone.0108716)

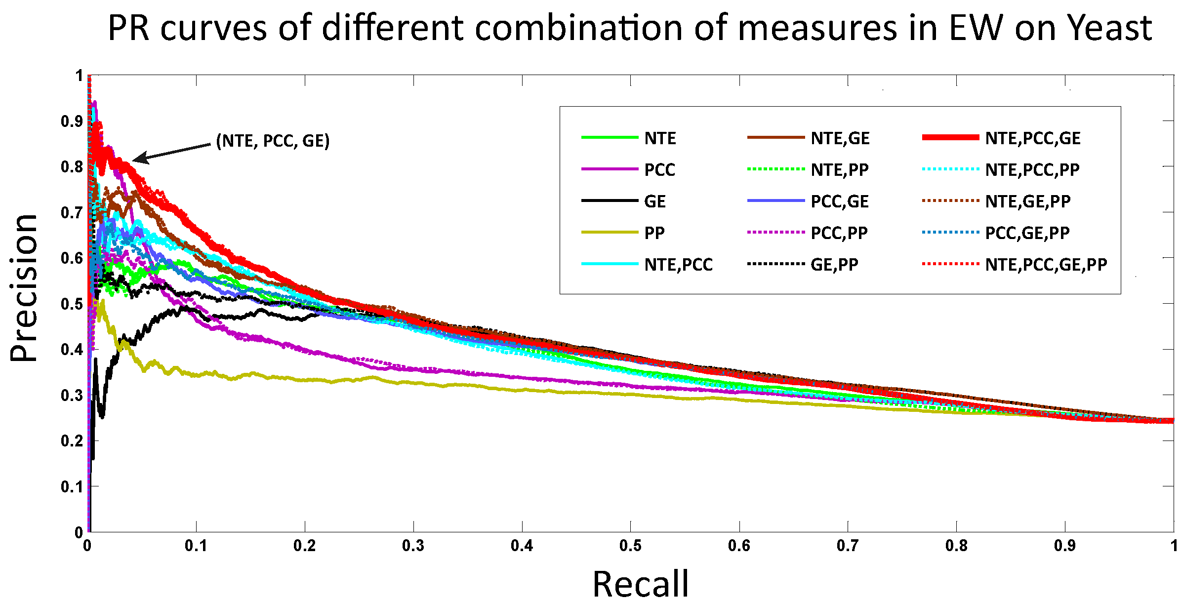

Supplement: Figure S1 — PR curves of different combination of measures in EW on Yeast PPI networks. 15 PR curves with different combination of of in formula (1) are illustrated. It can be seen that the results using the combination , which is the top red line above all the others, has the best performance, which leads to as the formula (2) for the EW method. (TIF) [file pone.0108716.s001.tif]

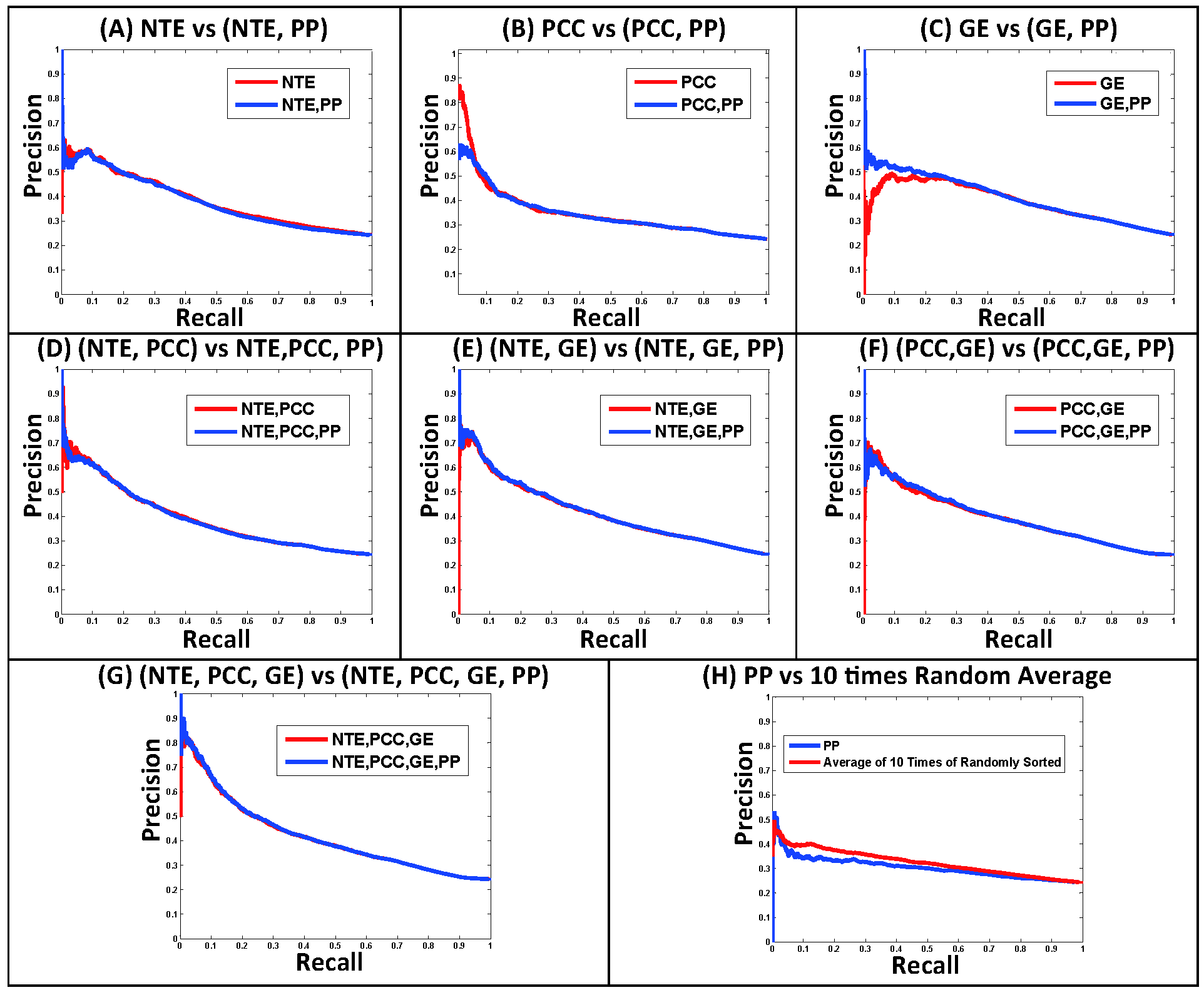

Supplement: Figure S2 — PR curves of different measures combination in EW without PP vs. with PP on Yeast PPI networks. And PP vs. average performance by 10 times randomly sorted all edges on Yeast PPI networks. From the PR curves in (A)–(G), we can see that PP has virtually no effect on to the combination identification, except when combined with GE. In (H), the performance with PP alone is very similar to the PR curve performance of randomly sorted all edges (by 10 times average). (TIFF) [file pone.0108716.s002.tiff]

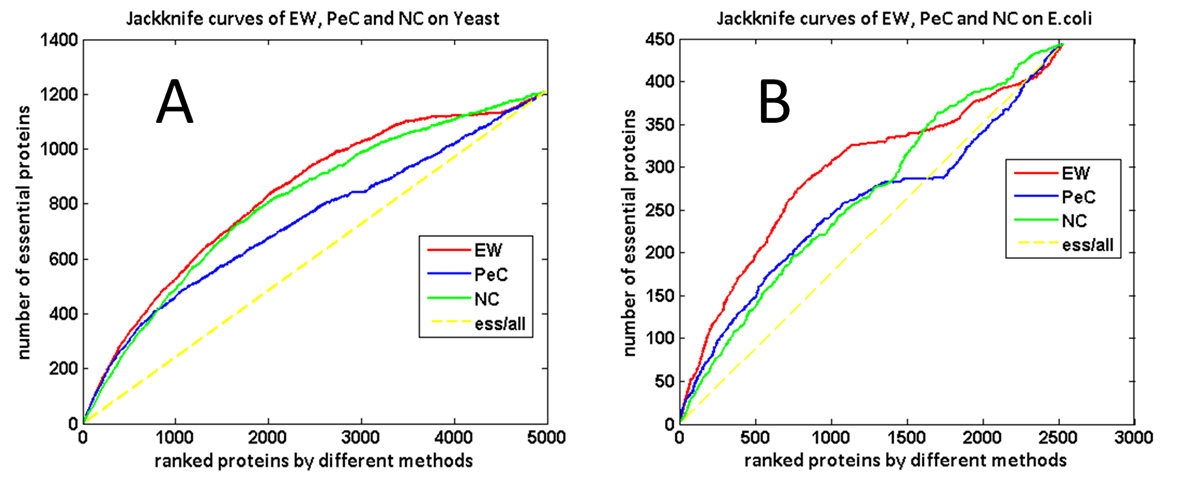

Supplement: Figure S3 — Jackknife curves of EW, NC and PeC on Yeast and E.coli PPI networks. The yellow line whose slope is equal to the ratio between the total number of essential proteins and the total number of all the proteins is plotted as a baseline. It represents the expected performance of the probability for a random selection that how many essential proteins will randomly appear in a chosen protein list and it is used as a standard reference for comparison. (TIF) [file pone.0108716.s003.tif]

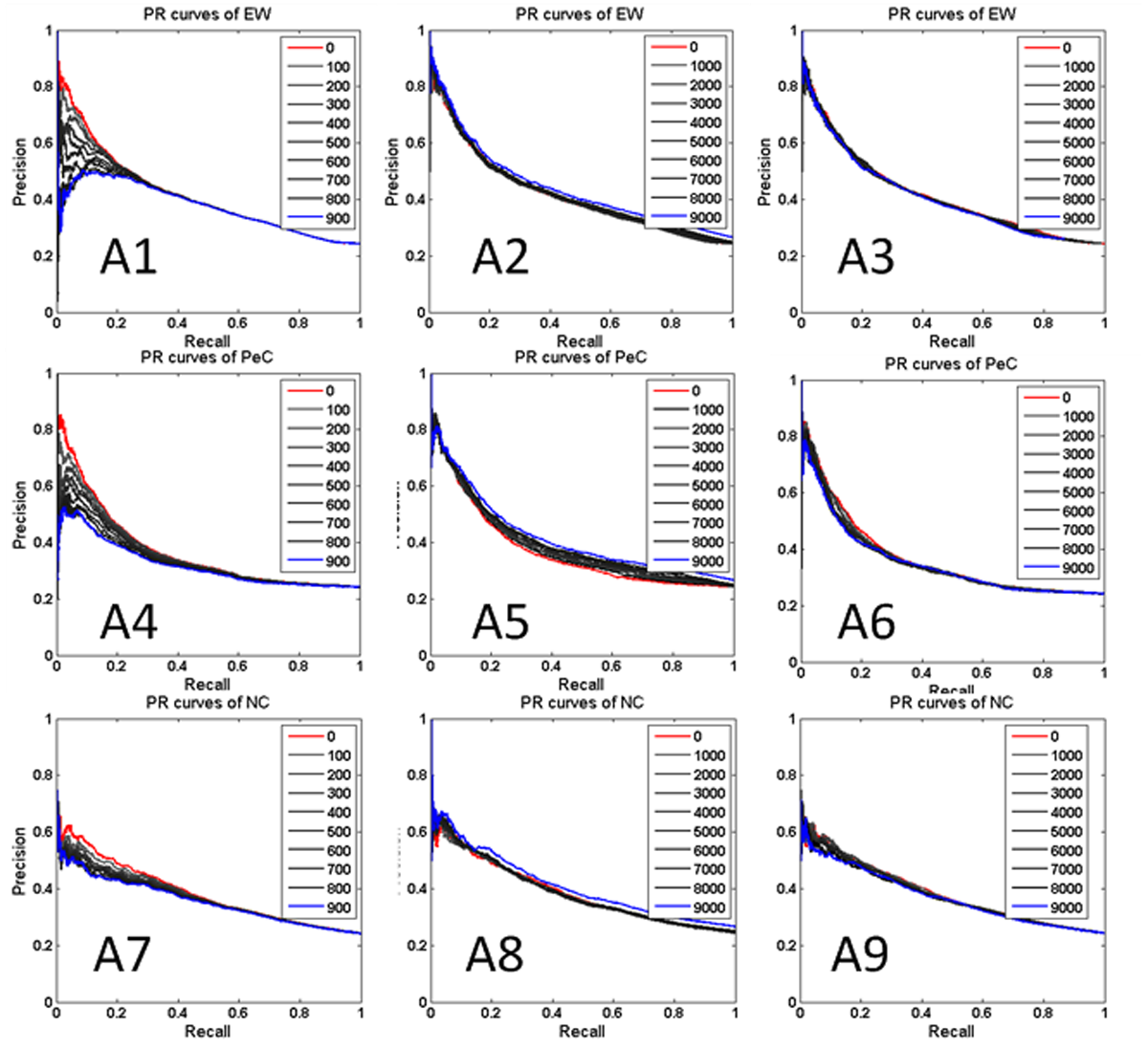

Supplement: Figure S4 — PR curves of EW, PeC and NC methods on the perturbed Yeast PPI networks for essential proteins identification. The Yeast PPI networks are perturbed from the top, the bottom and randomly by deleting X edges in 10 steps in the ranked EW edge lists. (A1, A4, A7) are the EW, PeC and NC performance of deleting edges from the top for X = 100; (A2, A5, A8) are the EW, PeC and NC performance of deleting edges from the bottom for X = 1000; (A3, A6, A9) are the EW, PeC and NC performance of deleting edges randomly for X = 1000. (TIF) [file pone.0108716.s004.tif]

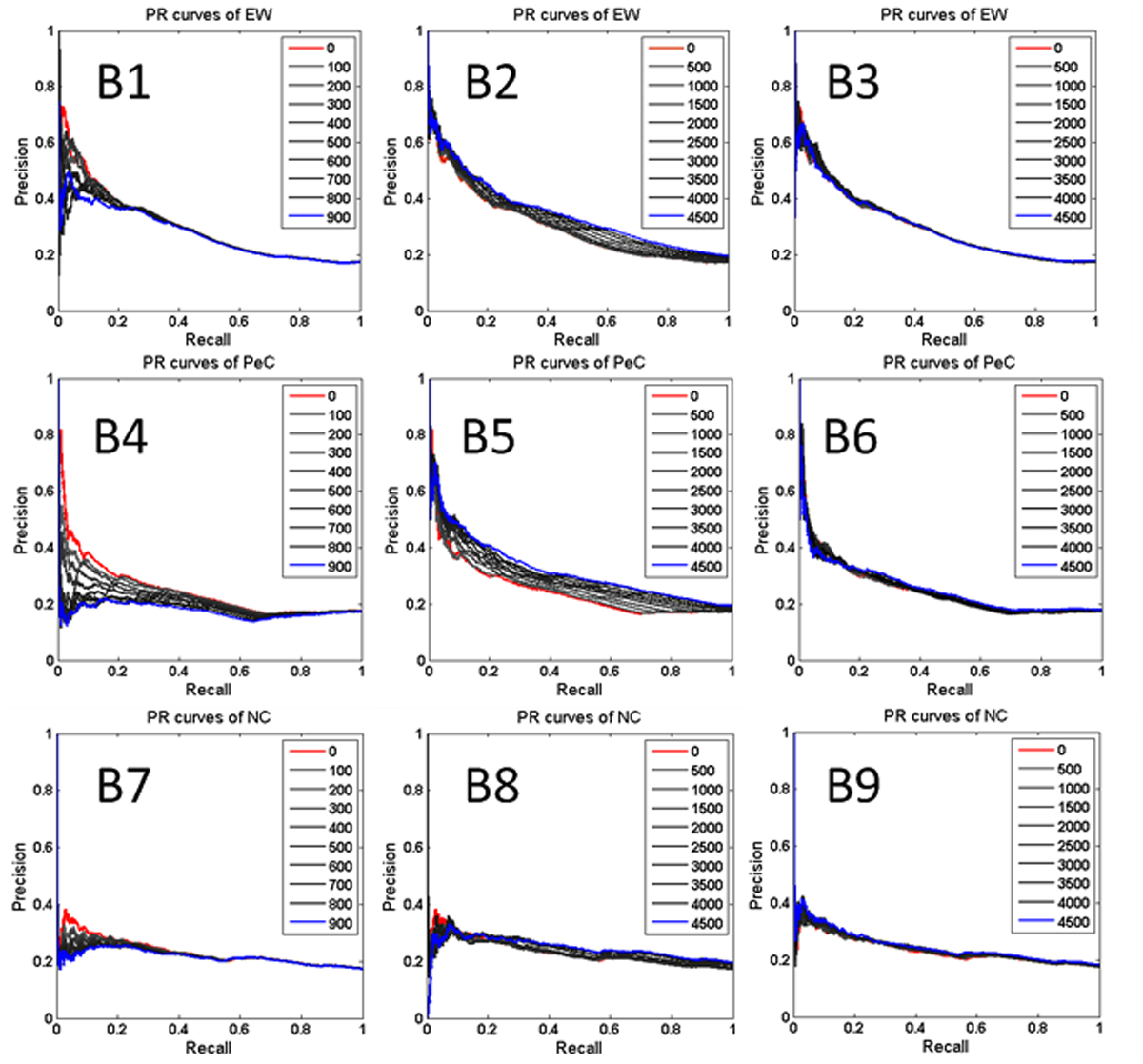

Supplement: Figure S5 — PR curves of EW, PeC and NC methods on the perturbed E.coli PPI networks for essential proteins identification. The E. coli PPI networks are perturbed from the top, the bottom and randomly by deleting X edges in 10 steps in the ranked EW edge lists. (B1, B4, B7) are the EW, PeC and NC performance of deleting edges from the top for X = 100; (B2, B5, B8) are the EW, PeC and NC performance of deleting edges from the bottom for X = 500; (B3, B6, B9) are the EW, PeC and NC performance of deleting edges randomly for X = 500. (TIF) [file pone.0108716.s005.tif]
