# Supplementary material for: Identification of Essential Proteins Based on Ranking Edge-Weights in Protein-Protein Interaction Networks
Source: PLoS One. 2014 Sep 30;9(9):e108716. doi: 10.1371/journal.pone.0108716 (PMC4182551; doi:10.1371/journal.pone.0108716)
Supplement: File S7 — Measures in Edge Weight. (DOC) [file pone.0108716.s012.doc]

**Measures in Edge Weight**

**Table of Content**

1. Number of Triangles an Edge Belongs
2. Gene Co-expression
3. GO Semantic Similarity
4. Pairwise Sequence Distance
5. **Number of Triangles an Edge Belongs**

Topological characteristics of PPI networks encode important information related to the lethality of the absence of a protein. Protein essentiality appears to be related on how many times a protein belongs to clusters that have cycles of odd numbered length, such as triangles and pentagons, and this information has been used in cluster and community detection methods [1] and centrality measures [2]. Yu *et al*. [3] determined that in an interaction network, essential proteins tend to be more cliquish. Estrada [4] proposed that protein indispensability does not depend on how close a protein is to many other proteins, nor on the number of protein-pairs a protein needs as intermediary in its communication along the protein-protein interactions. Instead Estrada reports that the proteins selected by any of the spectral measures of centrality form clusters of highly interconnected nodes showing a high number of triangles as measured by the clustering coefficient. Here we use the *Number of Triangles an Edge* (NTE) belongs to as one of the measures to identify essential nodes. To avoid degeneracy when the number of triangles is zero, we slightly modified the quantity by adding one.

In an undirected graph *G = (N, E)*, where *N* is the set of the proteins (nodes) in the network, and *E* is the set of the interactions (edges), the *NTE* of an edgeis defined as:

where (or $N_{v}$) is the set of neighbours of node *u* (or *v*) but do not include *u* (or *v*) itself, $\left|N_{u}\cap N_{v}\right|$ is the number of nodes in the intersection set of neighbour sets of and $N_{v}$, which is the number of triangles the edge (*u*, *v*) belongs to.

1. **Gene Co-expression**

Gene co-expression is increasingly used to explore the system-level functionality of genes. Studying co-expression patterns can provide useful insights into the underlying cellular processes, since the co-expressing genes could encode interacting proteins. Different measures for evaluating how significant two genes are co-expressed are widely accepted. In our method, we use Pearson Correlation Coefficient $PCC(u,v)$ as the co-expression measure of the pair proteins (and) interacting in the protein-protein interaction network [5].

where genes ( and ) encode the corresponding pair of proteins (and), *s* is the number of samples of the gene expression data; (or ) is the expression level of gene (or $V$) in the sample *i*; (or ) represents the mean of expression level of gene (or ), and (or ) represents the standard deviation of expression level of gene (or ).

1. **GO Semantic Similarity**

GO (Gene Ontology) [6] is designed to represent the known relationships between biological terms and the genes that are instances of those terms. GO semantic similarity is based on the biological characteristics of genes to reveal genes functionally similarity. Here we use Resnik algorithm [7] which is widely accepted and it is the default method in the GO tool FastSemSim, a package that implements several semantic similarity measures and provides an extensible set of classes that can be used to integrate semantic similarities into different analysis pipelines [8].

For finding significant GO terms between each pair of proteins, $GE(u,v)$ is defined as follows

where genes ( and ) encode the corresponding pair of proteins (and), is the probability of encountering an instance of concept *c*; is the set of concepts that subsume both and . We choose the maximum value of as $sim(U,V)$.

1. **Pairwise Sequence Distance**

Sequence distance is widely applied in phylogenetic and orthologous analysis. If two proteins tend to be similar in sequence, then they have small sequence distance and their biology function may be similar. In our method, we use the Jukes-Cantor distance [9] which is a commonly used method to score the sequence similarity in DNA, RNA and protein. The method assumes that each amino acid has the equal probability to change into other 19 kinds of amino acids and calculates the maximum likelihood estimate of the number of substitutions between two sequences. For protein andtheir Jukes-Cantor distance $PP(u,v)$ is:

$$PP(u,v)=-\frac{19}{20}*\log(1-\frac{20}{19}p)$$

where $p$ is the proportion of sites where the two sequences are different, is close to 1 for poorly related sequences, and is close to 0 for very similar sequences.

**References**

1. Radicchi, F., Castellano, C., Cecconi, F., Loreto, V. and Parisi, D. (2004) Defining and identifying communities in networks. Proc Natl Acad Sci U S A, 101, 2658-2663.
2. Wang, J., Li, M., Wang, H. and Pan, Y. (2012) Identification of essential proteins based on edge clustering coefficient. IEEE/ACM Trans Comput Biol Bioinform, 9, 1070-1080.
3. Yu, H., Greenbaum, D., Lu, H.X., Zhu, X. and Gerstein, M. (2004) Genomic analysis of essentiality within protein networks. RNA, 71, 817-846.
4. Estrada, E. (2006) Virtual identification of essential proteins within the protein interaction network of yeast. Proteomics, 6, 35-40.
5. Li, M., Zhang, H., Wang, J.x. and Pan, Y. (2012) A new essential protein discovery method based on the integration of protein-protein interaction and gene expression data. BMC Syst Biol, 6, 15.
6. Ashburner, M., Ball, C.A., Blake, J.A., Botstein, D., Butler, H., Cherry, J.M., Davis, A.P., Dolinski, K., Dwight, S.S., Eppig, J.T. et al. (2000) Gene ontology: tool for the unification of biology. The Gene Ontology Consortium. Nat Genet, 25, 25-29.
7. Resnik P. (1999) Semantic similarity in a taxonomy: an information-based measure and its application to problems of ambiguity in natural language. J Artif Intell Res, 11, 95-130.
8. M, M. FastSemSim. <http://sourceforge.net/p/fastsemsim/home/Home/>, unpublished.
9. Jukes, T.H. and Cantor, C.R. (1969) Evolution of protein molecules.
